# Rapid decline of Zika virus NS1 antigen-specific antibody responses, northeastern Brazil

**DOI:** 10.1007/s11262-020-01772-2

**Published:** 2020-06-15

**Authors:** Andres Moreira-Soto, Gilmara de Souza Sampaio, Célia Pedroso, Ignacio Postigo-Hidalgo, Beatrice Sarah Berneck, Sebastian Ulbert, Carlos Brites, Eduardo Martins Netto, Jan Felix Drexler

**Affiliations:** 1 Charité-Universitätsmedizin Berlin, Humboldt-Universität Zu Berlin, and Berlin Institute of Health, Institute of Virology, Berlin, Germany; 2 Universidade Federal da Bahia, Salvador, Bahia, Brasil; 3 Department of Immunology, Fraunhofer Institute for Cell Therapy and Immunology, Leipzig, Germany; 4 Fundação José Silveira, Salvador, Bahia Brasil; 5 German Centre for Infection Research (DZIF), Braunschweig, Germany; 6 Martsinovsky Institute of Medical Parasitology, Tropical and Vector-Borne Diseases, Sechenov University, Moscow, Russia; 7 Institute of Virology, Helmut-Ruska-Haus, Campus Charité Mitte, Charitéplatz 1, 10098 Berlin, Germany

**Keywords:** Zika virus, Flavivirus, Serology, Antigens

## Abstract

Zika virus (ZIKV) is a positive-stranded RNA virus within the *Flaviviridae* family. After decades of circulation in Asia, ZIKV was introduced to Brazil in 2014–2015, associated with a rise in congenital malformations. Unlike the genetically related dengue virus (DENV), ZIKV constitutes only one serotype. Although assumed that ZIKV infection may engender lifelong immunity, the long-term kinetics of ZIKV antibody responses are unclear. We assessed long-term kinetics of ZIKV NS1-IgG response in 144 individuals from 3 different subpopulations: HIV patients, tuberculosis patients and healthy individuals first tested in 2016 and retested 1.5–2 years after the 2015–2016 ZIKV epidemic in Salvador de Bahia, Brazil, using a widely distributed NS1-based commercial ELISA. The seropositivity in 2016 reached 59.0% (85/144, 95% confidence interval (CI) 50.7–66.7%), and decreased to 38.6% (56/144, CI 31.3–47.0%) 1.5–2 years later. In addition, the median ZIKV NS1-ELISA reactivity for individuals that remained positive in both timepoints significantly decreased from a ratio of 4.4 (95% CI 3.8–5.0) to 1.6 (95% CI 1.6–1.9) over the 2-year interval (*Z*: − 6.1; *p* < 0.001) irrespective of the subpopulation analyzed. Initial 2016 DENV antibody response was non-significant between groups, suggesting comparable DENV background. The high 20.6% seroreversion suggest that widely used serologic tests may fail to account a considerable proportion of past ZIKV infections in flavivirus endemic countries. In addition, ZIKV immunity might be shorter-lived than previously thought, which may contribute to local ZIKV resurgence once individual immune responses wane sufficiently to reduce community protective immunity in addition to birth and migration.

## Introduction

The Zika virus (ZIKV) is an enveloped positive-stranded RNA virus belonging to the *Flavivirus* genus inside the *Flaviviridae* family. Unlike the ubiquitous dengue virus (DENV), which occurs as four distinct serotypes globally, ZIKV represents only a single serotype to which both the African and the Asian lineages of ZIKV belong [1, 2]. The ZIKV genome encompasses about 10.7 kb containing two non-coding regions (5′- and 3′-UTR) and a single open reading frame that encodes for a polyprotein subsequently cleaved into three structural (core, envelope and membrane precursor) and seven non-structural (NS1, NS2A, NS2B, NS3, NS4A, NS4B, NS5) proteins [3]. Virologic diagnosis usually requires both molecular detection and serologic detection of IgM and IgG antibodies, since viremia is usually low and transient [4]. ZIKV serologic diagnosis is mostly based on antibodies against two viral proteins, envelope and NS1 [5]. The envelope protein has critical roles in the assembly of virions and cell entry [6] and NS1 is a non-structural glycoprotein that plays a putative role in viral replication, and when secreted modulates viral immune invasion and pathogenesis [7]. The NS1 of flaviviruses contains more highly diversified epitopes than the envelope protein, therefore its wide use in flavivirus serologic tests [8].

ZIKV was first detected in 1947 in Uganda [9]. Later in 2007, ZIKV emerged in the Pacific island of Yap, in 2013 in French Polynesia and other Pacific islands and from there expanding to mainland Latin America in 2015 causing the biggest outbreak to date [10–12]. The limited serologic surveys that are available found a high-level population exposure reaching from 42% in French Polynesia and 49% in Martinique, to as much as 63% in mainland America, specifically Brazil [5, 13, 14]. If ZIKV confers long-lasting immunity, high exposure could create sufficient herd immunity limiting local resurgence and upcoming epidemics [5]. However, isolated island populations might not be comparable to mainland America. The Pacific islands are a diverse region in which the combined population consists of approximately 2.3 million people and the island surface usually extends over a few thousand km^2^ only. In contrast, Brazil has 210 million inhabitants spread over 8 million km^2^ (latest *United Nations Population Division* estimates). In Brazil, as other Latin American countries, cocirculation of other flaviviruses such as DENV, Yellow fever virus, Bussuquara, Cacipacoré, Ilhéus, Rocio and Saint Louis encephalitis virus might elicit unique flaviviral antibody responses that impact ZIKV-specific antibody kinetics [15–17]. Nonetheless, long-term antibody kinetics of individuals infected with ZIKV in Brazil are largely unknown. Here, we conducted a prospective observational cohort study monitoring putative ZIKV circulation and antibody responses over time of individuals infected with ZIKV in the metropolitan region of Salvador, Brazil.

## Results and discussion

A total of 144 samples were taken from individuals on 2 occasions. The samples from the first timepoint correspond to a cross-sectional study conducted at the University Hospital Professor Edgard Santos (UHPES) in Salvador de Bahia, which is one of the biggest public hospitals in the region, between February and May 2016 during the end of the ZIKV epidemic [5]. Samples belong to three different subpopulations: immunologically stable HIV-positive patients and healthy individuals from the UHPES and treated tuberculosis patients from the José Silveira Foundation-Brazilian Institute for Investigation of Tuberculosis. These populations were selected due to their regular visits to the hospital, which was the only inclusion criterion for this study. The follow-up assessment was performed to the same subpopulations 1.5–2 years later (median 1.8, IQR 1.5–1.9 years), between August 2017 and February 2018, through new interviews and blood collections (IRB number 2.326.141). Follow-up serum samples were obtained from 28 patients on treatment for active pulmonary tuberculosis; 93 immunologically stable HIV-positive patients under antiretroviral therapy; and 23 healthy individuals. Samples from both timepoints were tested using a highly sensitive real time RT-PCR [18]. No sample tested positive by RT-PCR. Although there was no RT-PCR confirmation of acute ZIKV infection, it is likely that ZIKV antibody responses are largely comparable between study participants, since all of them were likely infected in a very similar time span during 2015–2016, due to the ultra-rapid ZIKV spread in Salvador, northeastern Brazil [5].

**Fig. 1:**
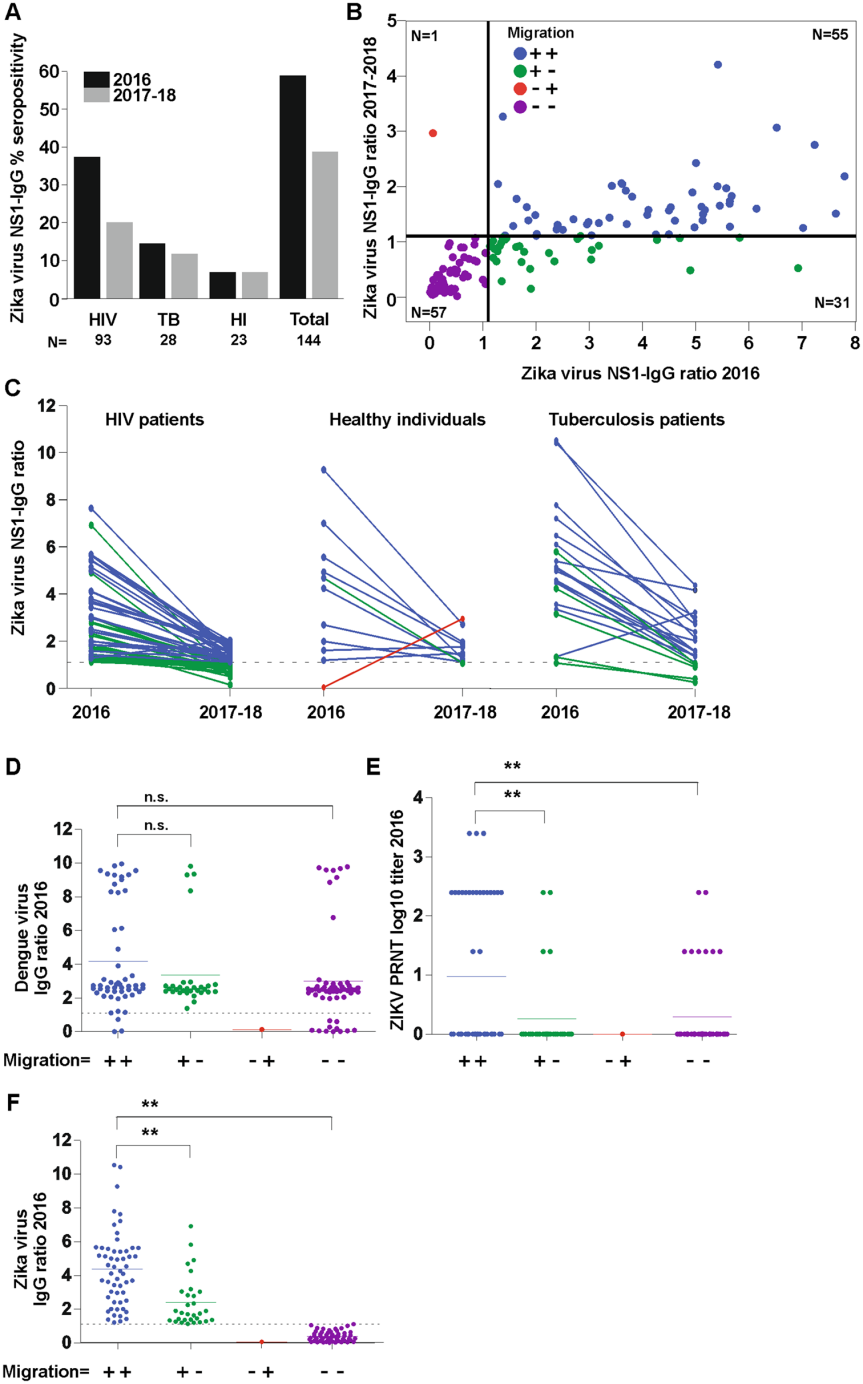
**a** Zika virus NS1-IgG seropositivity in 2016 (shown in black) and in 2017–2018 (shown in gray) in the HIV patients (HIV), tuberculosis patients (TB), healthy individuals (HI) subpopulations and total patients analyzed for the prospective study. **b** Comparison between the ZIKV NS1-specific IgG ratios during the epidemic (2016) and follow-up (2017–2018). Heavy lines (horizontal and vertical) correspond to the positivity cut-off ratio of 1.1 suggested by the manufacturer. Diagonal line divides the results among positive individuals in both time points who had an increase in the ratio of the follow-up sample (above) compared to the first one and the individuals whose value of the ratio decreased (below). **c** Zika virus NS1–IgG ratio per subpopulation. Colors as in **b**. **d** Dengue IgG ratios in 2016 between patients that remained positive in both timepoints (++), patients that seroreverted (+−), patients that seroconverted (−+) and patients that remained negative in both timepoints (–). Colors as in **b**. *n.s.* not significant. **e** Zika virus specific plaque reduction neutralization test (PRNT). Double asterisk denotes *p* < 0,001. Colors as in **b**. **f** Zika virus IgG ratios in 2016. Colors as in **b**. Double asterisk denotes *p* < 0,001

Brazil acquired millions of ZIKV NS1 antigen-based indirect ELISA tests (Euroimmun, Lübeck, Germany) for serological testing in public health laboratories [19]. We used the same NS1-based ELISA to compare detection between the paired serum samples from 2016 [5] and 2017–2018. The ZIKV seropositivity in the first timepoint in 2016 reached 59.0% (85/144, 95% confidence interval (CI) 50.7–66.7%) in concordance with the 63% seroprevalence from a serologic survey performed at the UHPES in 2015–2016 (Fig. 1a) [5]. Moreover, this seropositivity later decreased to 38.6% (56/144, CI 31.3–47.0%) 1.5–2 years later (Fig. 1a). Of the 59 ZIKV-negative individuals in 2016, only 1 individual belonging to the healthy individuals subpopulation seroconverted 2 years later, consistent with near-complete lack of ZIKV activity in northeastern Brazil after the large initial outbreak [5]. As shown in Fig. 1b, c, median ELISA reactivity for seropositive individuals that remained seropositive in 2017/2018 decreased significantly from a ratio of 4.4 (the ratio is built by dividing sample reactivity by the reactivity of a calibrator provided in the kit; 95% CI 3.8–5.0) to 1.6 (95% CI 1.6–1.9) over the 2-year interval (positivity threshold of the kit is a ratio of 1.1.; *Z* − 6.1; *p* < 0.001) irrespective of which subpopulation was analyzed. Among the initially seronegative individuals, median ELISA reactivity was almost identical between both time points (0.36–0.37; *Z* − 0.1; *p* = 0.88) (Fig. 1b). Remarkably, 30 individuals (20.6%; 95% CI 14.9–28.4) that were seropositive in 2016 were seronegative for ZIKV in 2017–2018, the majority of which (*n* = 25; 83.3%) were HIV-positive individuals (Fig. 1a, c). The level of seroreversion did not differ significantly among HIV-positive and tuberculosis patients [25 serorevertants (26.9%) versus 5 serorevertants (17.8%); Fisher, *p* = 0.23], whereas seroreversion occurred significantly less frequently in healthy individuals (1 serorevertant; 4.3%) than in HIV-positive patients (Fisher, *p* = 0.03) (Fig. 1c).

The high level of seroreversion can be explained by three scenarios. First, seroreversion in HIV-positive individuals may be associated with lower magnitudes of ZIKV-specific immune responses compared to other subpopulations, consistent with generally lower antibody responses of HIV-positive individuals, e.g., following hepatitis A virus vaccination [19]. However, the decrease of ELISA ratios in all analyzed subpopulations speaks against this scenario (Fig. 1c). Second, cross-reactive antibodies elicited by prior infection with flaviviruses other than ZIKV might have elicited false-positive ZIKV results during first testing. Cross-reactivity of dengue-specific IgG with ZIKV NS1 antigen has been well documented, ranging from 83.3% 3–6 months to 28.1% 1–2 years after secondary dengue infections [20]. If this was the case, patients that are ZIKV antibody positive in both timepoints might show different DENV-reactive antibody responses than serorevertants and participants that were ZIKV-negative in both timepoints. However, our data showed no difference in a DENV-reactive ELISA between the groups in 2016, suggesting comparable exposure to the hyperendemic DENV (Fig. 1d). Unfortunately, due to low sample volumes, no further test of the DENV antibody response in 2018 was performed. Next, to assess the specificity of the observed ZIKV reactive antibody responses, samples were analyzed by plaque reduction neutralization assays (PRNT) as previously described (Fig. 1e) [5]. In total, 30.0% (24/85) of the ZIKV NS1-ELISA-positive participants in 2016 where positive by PRNT. Compared to ELISA, PRNT is more specific, but also less sensitive [4, 17]. We observed that patients that seroreverted also had significantly lower PRNT titers (Fig. 1e) and ZIKV NS1-ELISA ratio (Fig. 1f) at enrollment than patients that remained positive in both timepoints. On the one hand, this may imply unspecific ELISA results at enrollment, hypothetically due to differences in the time since exposure to the hyperendemic DENV [21]. On the other hand, it seems unlikely that hypothetically unspecific results should not have occurred in the sera sampled 2 years later during 2018, because DENV immune responses are usually long-lasting [17, 20]. It thus seems likely that higher PRNT titers and ELISA ratios in study participants that remained positive over time represent stronger immune responses. The last explanation for our data could be that the lower responses observed may be limited to NS1-specific antibodies, which are elicited in infected individuals at much lower magnitude than antibodies against the ZIKV envelope antigen that also remain elevated for longer periods of time [22].

Irrespective of the complex underlying reasons, the observed decrease in ZIKV NS1-specific antibody levels over time was reminiscent of the complete lack of seroconversion in follow-up samples of two different cohorts. First, from RT-PCR-confirmed ZIKV infections in travelers using the same NS1-based serologic test 42 days post onset of symptoms [23]. Second, from a recent serological survey in the Pacific islands following patients during a 2-year period, showing a marked decrease in ZIKV overall seroprevalence in French Polynesia from 37 to 22% and in Fiji from 24 to 12% [24]. This decrease was observed for ZIKV and not for DENV, which causes sporadic outbreaks in the Pacific [24]. Similarly, DENV NS1-specific IgG antibodies were readily detected up to three years post-infection in Brazilian patients [20]. Those data are not at odds with our results, because DENV seroprevalence is as high as 80% in northeastern Brazil [5] and individuals frequently have multiple DENV infections likely boosting NS1-specific immune responses compared to ZIKV, which exists as a single serotype.

Our serologic data from Brazil are thus consistent with data from Pacific island populations in showing loss of ZIKV-specific antibody responses over a comparably short time span irrespective of the serologic method used for testing [24]. Since NS1-based serologic tests are widely used, it is possible that future seroprevalence studies will underestimate ZIKV spread, particularly in immunocompromised populations. This could also be the case for Africa, where despite the recent introduction of the Asian lineage from Latin America potentially causing congenital malformations, seroprevalence is several orders of magnitude lower than the observed for the Pacific islands or Latin America [22]. The combination of NS1-specific antibodies with other antigens and tests may be necessary to increase the reliability of future seroprevalence studies [4]. The relevance of an adequate determination of the flaviviral serostatus is illustrated by the interaction between DENV and ZIKV antibodies that may both protect from and enhance subsequent infections [21, 25], and by the growing number of flaviviruses that cocirculate in Brazil, for which their potential immune interplay is largely unknown [16, 17]. Since Brazil has licensed a DENV vaccine, adequate determination of the ZIKV serostatus and its potential interplay with DENV vaccination is crucial. Finally, studies analyzing long-lasting ZIKV-specific immune response are needed, as ZIKV immunity in flavivirus endemic countries might be shorter than previously thought. Decreasing individual-level immune responses in addition to the replenishment of susceptible individuals by birth and migration may sufficiently reduce community protective immunity to allow ZIKV resurgence.
